# Detection and Genetic Characterization of Border Disease Virus (BDV) Isolated from a Persistently Infected Sheep in a Migratory Flock from Rajasthan State, Northwestern India

**DOI:** 10.3390/v16091390

**Published:** 2024-08-30

**Authors:** Semmannan Kalaiyarasu, Katherukamem Rajukumar, Niranjan Mishra, Shashi Bhusan Sudhakar, Vijendra Pal Singh

**Affiliations:** ICAR-National Institute of High Security Animal Diseases, Anand Nagar, Bhopal 462022, India; krajukr@yahoo.com (K.R.); mishranir@rediffmail.com (N.M.); sudha.vet@gmail.com (S.B.S.); vijendra61@gmail.com (V.P.S.)

**Keywords:** border disease virus, pestivirus, genetic typing, persistent infection, migratory sheep

## Abstract

Border disease virus (BDV) causes significant economic losses in sheep farming worldwide. In India, BDV has not yet been studied in sheep migrating for summer pasturing. This study aimed to determine the extent of BDV infection in migratory sheep and provide genetic characteristics of BDV. Blood and serum samples from 90 lambs of a migratory sheep flock (600) in Central India were collected and subjected to molecular detection, phylogenetic analysis and virus neutralization test (VNT). We detected BDV in two lambs through real-time RT-PCR, while 64.4% (58/90) of in-contact lambs had BDV neutralizing antibodies. One apparently healthy lamb was found to be persistently infected with BDV. Phylogenetic analysis of 5′-*UTR* and *N^pro^* genes and the concatenated datasets typed the BDV isolate from PI sheep as BDV-3 genotype. However, it showed a closer relationship with BDV-3 strains from China than the previously reported Indian BDV-3 strains. This is the first report on the detection of BDV persistently infected migratory sheep in India. Additionally, we provided evidence of genetic variability among BDV-3 strains in India. The findings improve our understanding of epidemiology and genetic characteristics of BDV in India and highlight the potential risks associated with the traditional practice of sheep migration for summer pasturing.

## 1. Introduction

Border disease (BD), primarily a viral disease of sheep and occasionally of goats, is prevalent in most sheep-rearing countries worldwide. It causes significant economic losses in sheep farming due to congenital disease and reproductive disorders. Moreover, highly fatal BD epidemics causing a dramatic decrease (80%) in Pyrenian chamois population in Spain [1,2] threaten the extinction of this wildlife species. BD is caused mainly by border disease virus (species *Pestivirus D*), but in some countries, where there is close contact between sheep and cattle, a similar disease process can also be caused by bovine viral diarrhea virus 1 (species *Pestivirus A*) and bovine viral diarrhea virus 2 (species *Pestivirus B*). BDV, BVDV-1 and BVDV-2 along with other pestiviruses belong to the genus *Pestivirus* in the family *Flaviviridae* [3]. Besides sheep and goats, BDV can infect a wide range of even-toed domestic (cattle, pig, alpaca and llama) and wild ungulates (reindeer, chamois, Pyrenean chamois, mouflon, European hare, wild boar and Alpine ibex) naturally [4]. Transmission of BD occurs via both direct contact and vertical transmission. Persistently infected (PI) sheep excrete large amounts of BDV throughout their life and are the main sources of infection for spread and transmission and, hence, identification and removal of PI sheep is critical for control of BD [5].

Similar to other pestiviruses, the BDV genome consists of a single-stranded positive-sense RNA of about 12.3 kb in length. A single open reading frame codes for four structural proteins (*C, E^rns^, E1* and *E2*) and seven to eight non-structural proteins (*N^pro^*, *p7, NS2-3, NS4A, NS4B, NS5A, NS5B*), and is flanked by 5′- and 3′-untranslated regions (*UTR*). Among them, 5′-*UTR*, and *N^pro^* genetic regions are most commonly used for genetic typing and phylogenetic analysis of BDV strains [6,7,8,9,10,11]. High genetic diversity among BDV strains has been reported and, so far, BDV strains have been classified into eight genotypes (BDV-1 to BDV-8), apart from Tunisian, Tunisia-like, Aydin-like and ovine pestivirus genetic groups, which cause BD-like syndromes [7,8,9,10,11,12,13,14,15].

India is home to about 74.2 million sheep and 148.8 million goats, and small ruminants contribute significantly to the agricultural economy in terms of production of meat, wool, hair, milk and manure as per the 20th Indian Livestock Census, 2019, DAHD, Government of India [15]. Sheep and goats are reared mostly by marginal and landless farmers to sustain their livelihood and nutritional security. The western state of Rajasthan accounts for about 9.5% of total sheep population in India, where livestock mobility is critical for local livelihood and due to the scarcity of fodder and water in arid regions, migrant pastoralists traditionally undertake temporary sheep migration to neighboring states for pasturing during summer, which carries the potential risk of spread and transmission of endemic and emerging sheep diseases [16]. In India, the first case of BDV infection was confirmed in sheep showing respiratory and reproductive disorders in Jammu and Kashmir State in 2010, and all eight BDV strains reported in the study were genetically typed as BDV-3 genotype [11]. Although genetic characterization of BDV isolates from different geographical regions is essential to improve knowledge on the epidemiology of BD, there is no report on the identification and genetic characteristics of BDV circulating in other regions of India and particularly in migratory sheep. Moreover, most studies on BDV genetic typing have so far been reported from countries in Europe, while there is very limited information on BDV genotypes circulating in South Asian countries. Here, we report the first identification of BDV persistently infecting migratory sheep in India and genetic characterization of the BDV involved.

## 2. Materials and Methods

### 2.1. Collection of Samples

As a part of pestivirus surveillance, samples were collected from sheep belonging to a migratory sheep flock located at Ganjbasoda village in the Vidhisha district of Madhya Pradesh State, in Central India. The sheep flock migrating from Rajasthan State in Western India had 600 native Indian meat breed (Marwari) sheep, comprising 460 adults and 140 lambs, and had travelled about 950 km along with the animal keepers. All the animals were apparently healthy during sampling, but the flock had a history of reproductive disorders including abortions in ewes as per the owner. Whole blood samples from a total of ninety 3–6-month-old lambs (about 15% of the flock) were randomly collected in vacutainers with and without EDTA for PBL and serum separation, respectively, and transported to the laboratory in ice. Additionally, resampling was carried out from two BDV RNA-positive sheep after one month, to confirm PI status. Peripheral blood leukocytes (PBLs) were separated from EDTA blood by density gradient centrifugation at 1000 rpm for 40 min using Histopaque (Histopaque-1077; Sigma, St. Louis, MO, USA) following the previously reported method [17], but with the modification that 0.8% ammonium chloride was used for lysis of the RBCs. Blood samples without EDTA was allowed to clot at room temperature and serum was separated by centrifugation at 5000 rpm for 5 min. Following separation, PBL and serum samples were stored at −80 °C until use. This study was approved by the Institute Animal Ethical Committee (No. 38/IAEC/HSADL/09) of ICAR-NIHSAD, Bhopal, India.

### 2.2. Pestivirus Detection and Differentiation by Real-Time RT-PCR

RNA was extracted from 140 μL of serum using a QIAamp viral RNA mini kit (Qiagen, Hilden, Germany), while RNA extraction from PBL was carried out using an RNeasy Mini kit (Qiagen, Germany), following the manufacturer’s protocols, and stored at −80 °C until use. To determine the extent of pestivirus infection, all the PBL and serum samples were initially subjected to a pestivirus generic TaqMan real-time RT-PCR targeting the 5′-*UTR* of the pestivirus genome using Light Cycler 480 (Roche, Indianapolis, IN, USA). The assay was conducted in 25 µL of reaction volume using the primers (BVD190-F, V326) and probe (TQ-Pesti) as described [18], and a Superscript III Platinum one-step real time RT-PCR reagent (Invitrogen, Carlsbad, CA, USA). Samples found positive for pestivirus RNA by the pestivirus generic TaqMan assay were then tested by differential TaqMan real-time RT-PCR in uniplex format using primers and probes specific to BVDV-1, BVDV-2, HoBiPeV and BDV; Light Cycler 480; and the SuperScript III Platinum One-Step Quantitative RT-PCR system (Invitrogen, USA), as reported previously [19,20,21].

### 2.3. Pestivirus Antigen Detection

The blood leukocytes were tested for the presence of pestivirus antigen by a commercially available E^rns^ mAb-based antigen ELISA kit (IDEXX BVDV Ag/Serum Plus, IDEXX, Baar, Switzerland), following the manufacturer’s instructions.

### 2.4. Virus Isolation and Identification

Pestivirus-free fetal sheep thymus cell line ‘SFT-R’ (RIE43), obtained from Cell Culture Collection of Veterinary Medicine, Friedrich-Loeffler Institute, Island of Riems, Germany, was used for virus isolation. The cells were maintained in Eagle’s Minimum Essential Medium (EMEM; Sigma) containing 10% horse serum (Gibco-BRL Life Technologies, Carlsbad, CA, USA) at 37 °C in 5% CO_2_ atmosphere. Blood leukocytes and serum samples from BDV RNA-positive sheep were inoculated onto sub-confluent monolayers of SFT-R cells in 25 cm^2^ tissue culture flasks. After 5 days, the cultures were frozen and thawed thrice and the clarified supernatant was subjected to IPMA using a pool of pan-pestivirus reacting monoclonal antibodies (mAbs) WB103/WB105 (Veterinary Laboratory Agency, Weybridge, UK), as described previously [22]. RNA extraction was carried out from 140 µL of infected MDBK cell culture supernatants using a QIAamp viral RNA mini kit (Qiagen, Germany) and identification of the BDV was carried out by differential real-time RT-PCR assays, as described above. Antigenic typing of BDV isolated from PI sheep was performed by immuno-peroxidase monolayer assay (IPMA) using BDV-specific Mabs, as described previously [11].

### 2.5. BDV Antibody Detection

BDV antibody detection was performed on serum samples (*n* = 90) from sheep using a standard virus neutralization test (VNT) coupled with detection by IPMA following our previously described procedure [22]. Virus neutralization tests were performed in 96-well culture plates in triplicate wells using 1:5 dilution of heat inactivated serum, SFT-R cells and 200 TCID_50_ of Indian BDV isolate Ind 830-09 [11]. The serum, with a neutralizing titer of 1:10 against BDV, was considered to be BDV antibody-positive [23]. Serum samples from two BDV RNA-positive sheep collected after 1 month were tested again for the presence of BDV antibodies.

### 2.6. Confirmation of PI Status

Based on the results of Real-time RT-PCR from the first sampling, two male lambs (Sheep #12 and Sheep #13) were found to be infected with BDV. To confirm the status of BDV infection (acute or persistent infection), whole blood and serum samples were collected again after 1 month and subjected to BDV specific real-time RT-PCR, virus isolation, PACE and VNT. Subsequently, the PI lamb (Sheep #12) was procured from the animal owner and has been the subject of another study on the evaluation of an improved BDV real-time RT-PCR on manually plucked hair samples [24].

### 2.7. RT-PCR Amplification

Amplification of part of 5′-*UTR* (288 bp) of the pestivirus genome was carried out for the BDV-positive original clinical samples (blood leukocytes) and BDV isolates obtained in this study in a single-step RT-PCR using 324 and 326 primers [25] and Access RT-PCR kit (Promega, Madison, WI, USA). For amplification of the complete *N^pro^* gene of the BDV isolate, the cDNA synthesis was performed in 20 µL volume, as previously described [22], which was then used as a template in PCR. The PCR was carried out using the primers, BD1 and BD2 [26], to amplify a 738 bp fragment (position in BDV-1 strain X818: nt 354-1085) covering the entire *N^pro^*, and part of the C gene.

### 2.8. Nucleotide Sequencing and Sequence Analysis

The DNA fragments of 5′-*UTR* and *N^pro^* were purified by a QIAquick gel extraction kit (Qiagen) and were directly sequenced in both directions using gene-specific primers, an ABI PRISM Big Dye Terminator V.3.1 Cycle Sequencing Kit (Applied Biosystems, Foster City, CA, USA) and an ABI 3130 genetic analyzer (Applied Biosystems, USA) as per the manufacturer’s instructions. The partial sequences of 5′-*UTR* gene and full-length sequences of the *N^pro^* gene generated here have been deposited in NCBI GenBank under accession numbers ON227056 and ON227057, respectively. Additional sequences representing all genotypes of BDV and other reference pestiviruses were retrieved from NCBI GenBank and used for comparative sequence analyses. Sequence analysis was carried out for 243 nt of 5′-*UTR* and 504 nt of complete *N^pro^* sequences. The percentage of nucleotide identity values for both 5′-*UTR* and *N^pro^* and amino acid identity values for *N^pro^* were generated by MegAlign program of DNASTAR (Lasergene Inc., Madison, WI, USA). Nucleotide sequence alignment was performed using Clustal W in MEGA v7.0 [27]. MEGA v7.0 [27] was used for phylogenetic analyses and maximum-likelihood (ML) trees were constructed using the Kimura-2 parameter model. The robustness of the phylogenetic relationships was evaluated using bootstrap analysis with 1000 replicates.

## 3. Results

### 3.1. Identification of BDV-Infected Sheep

In this study, testing of whole blood and serum samples of sampled sheep (n = 90) by pestivirus generic TaqMan real-time RT-PCR showed that blood leukocyte samples of two sheep (Sheep #12, Sheep #13) and a serum sample of one sheep (Sheep #12) were positive for pestivirus genomic RNA. Differential TaqMan real-time RT-PCR results showed that both blood leukocyte and serum samples of Sheep #12 and the blood leukocyte sample of Sheep #13 were found positive for BDV, but were negative for BVDV-1, BVDV-2 or HoBiPeV. Of the two BDV-infected sheep, one (Sheep #12) was a 6-month-old male of a mutton breed with stunted body growth, while the other (Sheep #13) was a 7-month-old male of a mutton breed with normal body growth. Pestivirus antigen ELISA results revealed that blood leukocytes of only 1 (Sheep #12) of the 90 sheep tested positive (weak positive, just above the positive cut-off value) for pestivirus antigen. To determine the BDV antibody prevalence status, all 90 serum samples were tested by VNT and the results showed that 58 (64.4%) out of 90 sheep had neutralizing antibodies against BDV. Of the two BDV RNA positive sheep, one (Sheep #12) was negative, while the other one (Sheep #13) was positive for BDV neutralizing antibodies (BDV antibody titer 1:20).

### 3.2. BDV Isolation and Identification

When propagated on SFT-R cells, pestivirus was isolated from both blood leukocytes and serum of only one (Sheep #12) of the sheep, not only from the first sampling but also from second sampling done after 1 month. The pestivirus isolate (Ind 293299/12) was of ncp biotype and was identified as BDV by differential TaqMan real-time RT-PCR. The pestivirus isolate was also classified as BDV antigenically, as it could be neutralized by higher dilution of BDV polyclonal serum than BVDV-1 or BVDV-2 serum (>16-fold difference in titer) and showed strong reactivity with BDV-specific mAbs, but no reactivity with BVDV-1- or BVDV-2-specific mAbs. 

### 3.3. Identification of PI Sheep

Based on the results of Real-time RT-PCR, antigen ELISA, virus isolation and VNT for all 90 sheep from the first sampling and 2 sheep resampled after 1 month, 1 sheep (Sheep #12) was confirmed as PI, as it was found to be viremic (positive for Real-time RT-PCR, virus isolation and antigen ELISA) and was negative for BDV-neutralizing antibodies at both sampling times. Conversely, the other BDV RNA-positive sheep (Sheep #13) was confirmed as acutely infected, as it tested negative for virus isolation and antigen ELISA at the second sampling, and was positive for BDV-neutralizing antibodies at both sampling times.

### 3.4. Genetic Analysis of BDV in 5′-UTR

**Figure 1:**
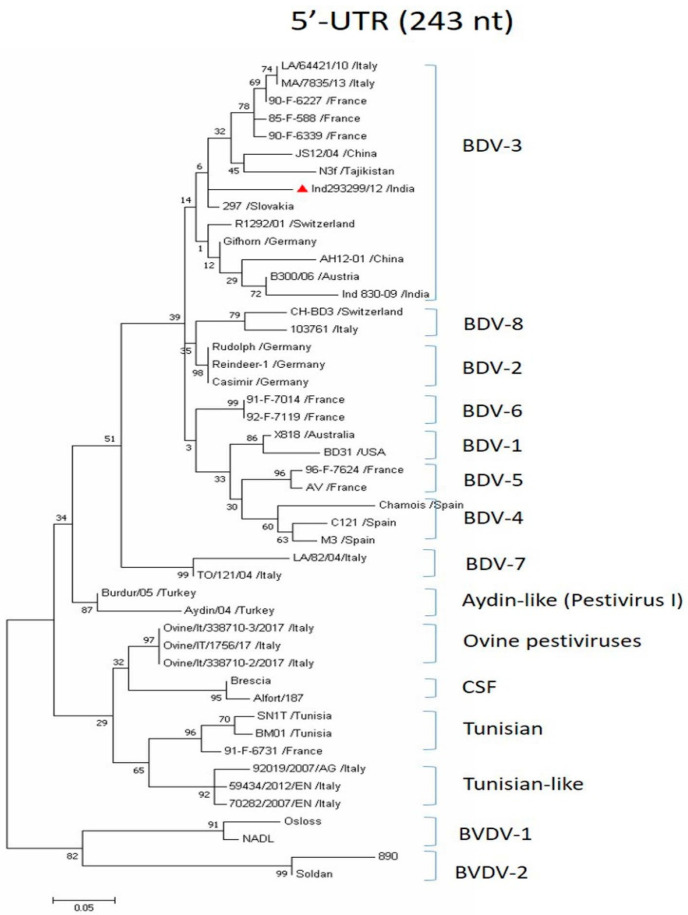
Phylogenetic tree based on 5′-*UTR* gene (243 bp). ML tree was prepared using MEGA v7.0. Numbers indicate the percentage of 1000 bootstrap replicates that support each phylogenetic branch. The sequence obtained in this study is labeled with a red triangle and other BDV sequences and pestiviruses analyzed are from GenBank.

Genetic analysis of partial 5′-*UTR* showed that the sequences obtained from original blood samples of both the PI sheep and the acutely infected sheep were almost identical, with 1–2 nucleotide differences indicating circulation of the same BDV strain. As *N^pro^* gene amplification was unsuccessful for the acutely infected sheep, genetic characterization of the BDV isolate (Ind 293299/12) from the PI sheep was conducted. Phylogenetic analysis of 5′-*UTR* sequences (243 bp) showed that the BDV isolate, Ind 293299/12, belonged to the BDV-3 genotype, but was more closely related to the BDV-3 strains from China (AH12-01, JS12/04), Slovakia (297) and Tajikistan (N3f) than the previously reported BDV-3 strain (Ind 830-09) from India (Figure 1). The nucleotide sequence identity with other BDV-3 strains ranged from 86.3% to 91.3%—the lowest with BDV-3 strains MA/7835/13/Italy and LA/64421/10/Italy from Italy and the highest with BDV-3 strain 297 from Slovakia—while it shared 75.8–90.9% nucleotide sequence identity with other BDV genotypes (Table 1). However, it showed 88.0% sequence homology with the previously reported BDV-3 strain (Ind 830-09) from India [11].

### 3.5. Genetic Analysis of BDV in N^pro^

**Figure 2:**
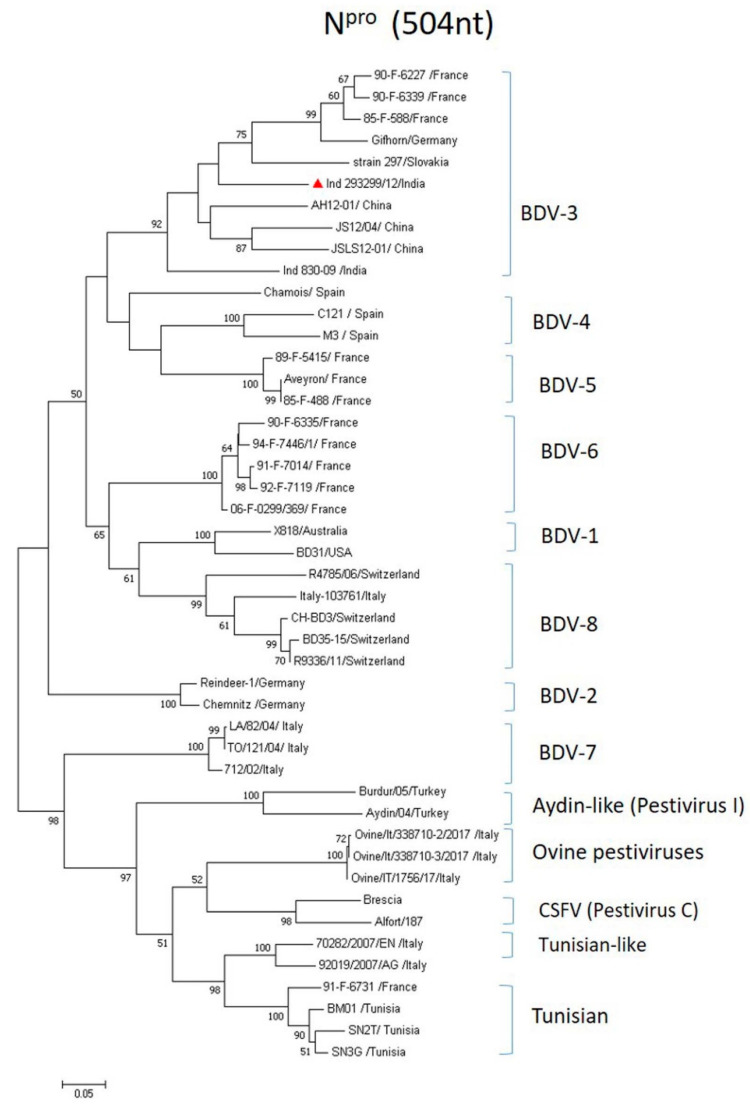
Phylogenetic tree based on sequences coding N^pro^ gene (504 bp). ML tree was constructed using MEGA v7.0. Numbers indicate the percentage of 1000 bootstrap replicates that support each phylogenetic branch. The sequence obtained in this study is labeled with a red triangle and other BDV sequences and pestiviruses analyzed are from GenBank.

To confirm the genotype of the BDV isolate from the PI sheep and assess its genetic relationship with other globally circulating BDV, we then analyzed the entire *N^pro^* gene. BDV-3 sequences from Austria, Switzerland, Italy and Tajikistan could not be included in the analysis due to unavailability of their *N^pro^* full-gene sequences in the public database. Phylogenetic analysis of *N^pro^* sequences (504 bp) revealed that BDV strain Ind 293299/12 belongs to the BDV-3 genotype, but was placed in a branch clearly separated from the previously reported Indian BDV-3 strain Ind 830-09 (Figure 2). When compared with other BDV-3 strains, Ind 293299/12 was found to be more closely related to the BDV-3 strains circulating in goats (AH12-01, JS12/04) and sheep (JSLS12-01) from China, while BDV-3 strains from Germany and France grouped together. There was only 78.6% nucleotide and 86.9% amino acid sequence homology between the Indian BDV-3 strains, indicating considerable genetic variability among them (Table 1). The nucleotide sequence identity of BDV Ind 293299/12 with other BDV-3 strains ranged from 76.6% to 81.5%—the lowest with BDV-3 strain Gifhorn from Germany and the highest with BDV-3 strain AH12-01 from China—while it shared 67.7–77.8% nucleotide and 73.2–83.9% amino acid sequence identity with other BDV genotypes.

### 3.6. Genetic Relationships of BDV in Concatenated 5′-UTR-N^pro^ Datasets

**Figure 3:**
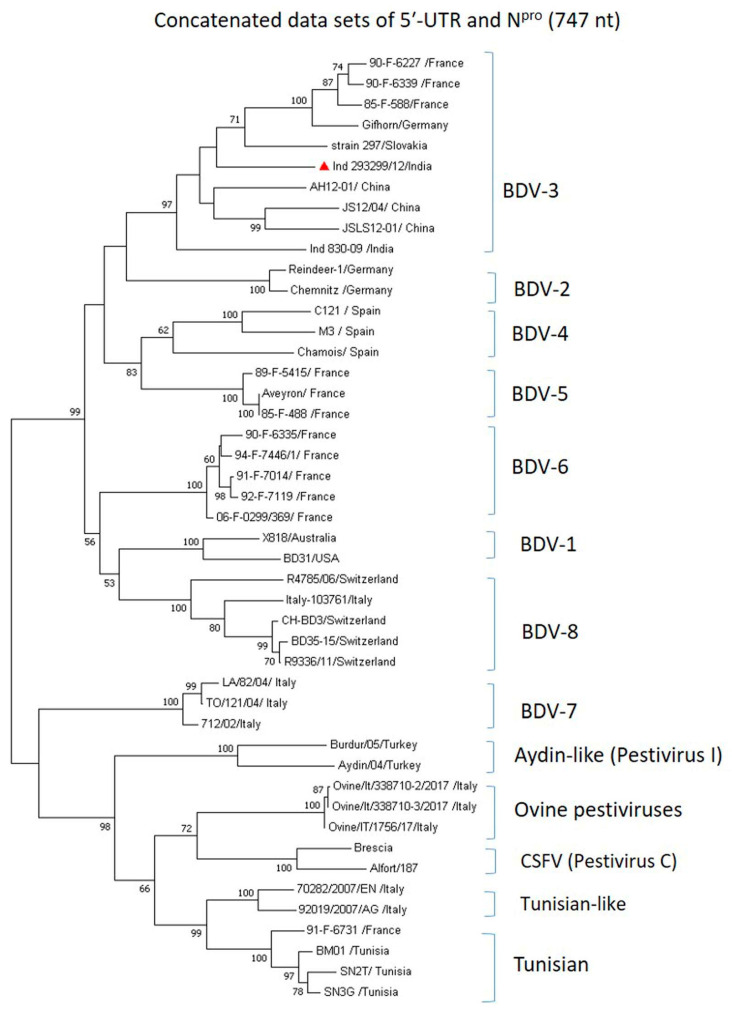
Phylogenetic tree based on concatenated 5′-*UTR*-*N^pro^* datasets (747 bp). ML tree was constructed using MEGA v7.0. Numbers indicate the percentage of 1000 bootstrap replicates that support each phylogenetic branch. The sequence obtained in this study is labeled with a red triangle and other BDV sequences and pestiviruses analyzed are from GenBank.

Additional phylogenetic analysis of BDV strains was carried out for the concatenated datasets (747 bp) of 5′-*UTR* and *N^pro^*. The results confirmed that BDV isolate Ind 293299/12 belongs to the BDV-3 genotype and the topology of the phylogenetic tree (Figure 3) was similar to that found for the *N^pro^* gene-based tree.

## 4. Discussion

This study describes detection of sheep persistently infected with BDV in a migratory sheep flock for the first time in India, indicating the possible risks of traditional practices of sheep migration by nomadic farmers in the epidemiology of border disease. Additionally, we show that the BDV strain found in the PI sheep belongs to the BDV-3 genotype, and is genetically more closely related to the BDV-3 strains circulating in China [28] than the previously reported BDV-3 strains in India [11].

In this study, BDV genomic RNA was detected in two lambs of the migratory sheep flock, while travelling through Madhya Pradesh State in Central India. BDV could be isolated from both blood and serum from one of the lambs (Sheep #12) on both sampling occasions (at a one-month interval), while it remained seronegative, confirming that this lamb was persistently infected with BDV, in concurrence with earlier studies [5,29]. This was also supported by the findings that a high proportion of the sampled in-contact sheep in the flock had BDV-neutralizing antibodies, consistent with a previous study [30]. The PI lamb was apparently healthy without any typical clinical signs of BD, emphasizing the challenges in clinical diagnosis of BD and requirement of laboratory diagnosis [5]. Similar findings have been reported previously showing that PI sheep shed BDV throughout their life and are major drivers of BD epidemiology and that a proportion of PI lambs may appear healthy [5,23,28]. However, the source of BDV infection could not be ascertained, as the dam of the PI lamb was not available for sampling and it had been sold by the owner about 4 months prior to sampling. The other BDV-infected lamb was found transiently infected, as evident by the presence of BDV RNA only on the first sampling.

In India, BDV infection has been detected only in the farmed sheep so far [11]. This study reports detection of BDV PI sheep in migratory sheep flock in India for the first time. Identification of migratory sheep persistently infected with BDV on a traditional migratory route in the plains (from Rajasthan State in western India through Madhya Pradesh State in Central India) has potential implications with regard to epidemiology of BD. Firstly, BDV PI sheep can infect naïve sheep, goat and cattle along the migratory route, as sheep, goat and cattle are commonly kept together and co-pasturing is a common practice among sheep and goat farmers, including nomadic sheep farmers, in India. Secondly, there is a risk of BDV transmission to cattle, as natural BDV infection in cattle has been reported in several sheep-rearing countries [8,29,31,32,33], following exposure with PI sheep during co-pasturing [34,35] or close contact. Thirdly, co-pasturing during migration such as alpine community summer pasturing in Switzerland has been found as a major risk factor for transmission of BDV from PI sheep to in-contact sheep and cattle [35]. Moreover, BDV PI sheep remain a high risk factor for BVDV-free cattle herds [36] and may thus impact BVD control programs. Although a limited number of samples were investigated here, the complex epidemiological features of BDV infection in susceptible hosts cannot be overlooked and more extensive studies are required to provide a better picture on the prevalence of BDV PI sheep and the extent of BDV infection in cattle in India.

The genetic diversity of BDV is greater than that of most other pestivirus species. To date, BDV strains within *Pestivirus D* species have been classified into eight genotypes (BDV-1 to BDV-8) [4,7,8,9,10,11,12,13,20]. Phylogenetic analysis based on the 5′-*UTR* and complete *N^pro^* gene sequences or the concatenated datasets typed the BDV isolate (Ind 293299/12) from the PI migratory sheep as BDV-3. So far, the BDV-3 genotype has been reported in sheep in eight countries—Germany [6], France [7], Austria [36], Switzerland [37], Italy [38], Slovakia [39], China [28] and India [11]—while BDV-3 partial 5′-*UTR* sequences are also available from Tajikistan (GenBank: KX900608). Previous studies have shown that BDV-3 is the most prevalent genotype in Europe. In Asia, BDV-3 has been detected in sheep and goats in China [28,40] and in sheep in India [11], while BDV-1 has been detected in pigs in Japan [41]. Detection of BDV-3 in migratory sheep from a different geographical region in India in this study provides evidence that this genotype may be the most prevalent BDV genotype also in Asia [11,28,40,42]. However, additional data from Asian countries are required to substantiate this observation.

Although seroprevalence studies are important for identifying exposure to pestiviruses in a flock, variable seroprevalence rates have been reported in small ruminants, depending on the geographic area. In India, a pestivirus seroprevalence rate of 23.4% in sheep and 16.9% in goats and a flock-level seroprevalence rate of 66.3% in sheep and 54% in goats have been reported previously [43]. In contrast, a high level of BDV seroprevalence (64.4%) in sheep was observed in this study, which is even higher than the 52.9% in sheep reported earlier [11]. Higher BDV seroprevalence rates than BVDV-1 or BVDV-2 have also been reported in Switzerland—56.1% (BDV-1) and 12.9% (BVDV-1) among sheep and 23.4% (BDV-1) and 10.2% (BVDV-1) among goats [44]. Varying rates of herd-level seroprevalence have been reported in different sheep- and goat-rearing countries, such as 30% in Poland [45], 89% in Austria [46], 25.81–41.77% in Italy [47], 75.9% in Turkey [9] and 68.2% in Algeria [48], whereas a low level of seroprevalence has been reported in Northern Ireland [49], Australia [50] and Greece [51].

There appears to be a geographical bias regarding the distribution of BDV genotypes, as BDV-1 has been identified in the United Kingdom [26], USA [52], Australia [53], New Zealand [26], Italy and Mexico [33,38]; BDV-2 in Germany [42]; BDV-3 in Germany, France, Italy, Austria, Switzerland, Slovakia, China, India and Tajikistan [6,7,11,28,36,37,38,39]; BDV-4 in Greece, Spain and France [1,52,54]; BDV-5 in France, Spain and Italy [7,38,55]; BDV-6 in France [7]; BDV-7 in Italy [38]; and BDV-8 in Italy and Switzerland [4,14,37]. On the contrary, prevalence of more than one BDV genotype has been reported in Italy, France, Germany, Spain and Switzerland. Hence, more genetic typing data from sheep-rearing countries around the world, including India, in the future may make this picture clear and improve our knowledge on the global epidemiology of BDV.

Here, we found a high genetic variability among the BDV-3 strains from India, as the BDV-3 strain from the PI sheep shared 88.0% (5′-*UTR*) and 78.6% (*N^pro^*) homology with the previously reported BDV-3 strains [11] and they branched separately, indicating at least two separate BDV introductions. Similar to our findings, high genetic diversity among BDV-3 strains has also been reported in China [28]. It is also noteworthy from the results of phylogenetic analyses in 5′-*UTR*, *N^pro^* or the concatenated datasets to find that BDV-3 strains from India (this study and the earlier report) are more closely related with BDV-3 strains from China, compared to those found in other countries, indicating a probable common origin. However, the exact source of origin of BDV in India is not yet known, as trade in live sheep of exotic origin has largely been restricted to Europe and Australasia, and there is no direct epidemiological link, as there has been no formal trade in sheep between India and China. However, the present findings emphasize the need for thorough vigilance of informal and illegal sheep trade along the Indo-Chinese border and implementation of strict sanitary measures during import.

In conclusion, BDV-persistent infection in migratory sheep was demonstrated for the first time in India, indicating the potential risks associated with the traditional practice of sheep migration in BDV transmission. Additionally, genetic characterization of the BDV extended our knowledge and insights on epidemiology and genetic diversity of BDV strains, highlighting the need for surveillance of BDV covering major sheep-rearing regions of India to optimize the control measures.

## Figures and Tables

**Table 1:** Homology between BDV isolate (Ind 293299/12) from PI sheep in this study and BDV strains representing all eight genotypes and other selected pestivirus strains in 5′-*UTR* (243 bp) and *N^pro^* (504 bp) gene.

S.No	Name of Reference Viruses	Genotype	Identity of Ind 293299_12 with Other Reference Viruses		
			5′-*UTR* (243 nt)	*N^pro^* (504 nt)	
			Nucleotide	Nucleotide	Amino Acid
1.	BD31	BDV-1	86.7	72.4	79.8
2.	X818	BDV-1	85.0	74.6	79.8
3.	Reindeer	BDV-2	87.5	76.6	81.5
4.	85-F-588	BDV-3	89.2	81.2	86.9
5.	AH12-01_China	BDV-3	90.9	81.5	90.5
6.	Gifhorn	BDV-3	88.7	76.6	81.5
7.	Ind830-09	BDV-3	88.0	78.6	86.9
8.	JS12-04_China	BDV-3	89.6	80.8	86.9
9.	JSLS12-01_China	BDV-3	82 (225 bp)	80.4	85.7
10.	Strain 297_Slovakia	BDV-3	91.3	80	84.5
11.	90-F-6339	BDV-3	88.4	81	86.3
12.	90-F-6227	BDV-3	88.0	80.4	86.9
13.	LA-64421-10	BDV-3	86.3	-	-
14.	MA-7835-13	BDV-3	86.3	-	-
15.	C121	BDV-4	85.1	73.6	82.7
16.	Chamois	BDV-4	85.1	75.6	81
17.	M3	BDV-4	86.3	74.6	81.5
18.	89-F-5415	BDV-5	85.9	75.2	79.2
19.	Aveyron	BDV-5	86.7	75	78.6
20.	90-F-6335	BDV-6	88.4	77	82.1
21.	91-F-7014	BDV-6	90.9	75.8	82.1
22.	92-F-7119	BDV-6	90.9	76.8	83.3
23.	06-F-0299-369	BDV-6	87.5	77.4	83.9
24.	94-F-7446-1	BDV-6	87.1	76.6	82.7
25.	712-02	BDV-7	82.8	71.6	81
26.	TO-121-04	BDV-7	87.1	72.4	81.5
27.	CH-BD3	BDV-8	86.7	77	81.5
28.	R4785-06	BDV-8	86.3	74	79.8
29.	R9336-11	BDV-8	84.4	77.8	82.1
30.	Italy-103761	BDV-8	85.9	75.4	81
31.	Burdur-05	Aydin-like	75.8	70	73.2
32.	Aydin	Aydin-like	78.5	67.7	73.2
33.	Ovine_IT_1756_17	Ovine pestiviruses	81.3	70	73.8
34.	Ovine_It_338710-2_2017	Ovine pestiviruses	81.3	70.2	73.2
35.	BM01	Tunisian	80.8	72.4	78.6
36.	SN2T	Tunisian	80.3	71	77.3
37.	92019-2007-AG	Tunisian-like	83.8	70.8	76.2
38.	70282-2007-EN	Tunisian-like	82.6	71.8	79.2
39.	Brescia (CSFV)	CSFV	80.8	68.1	73.2
40.	Alfort (CSFV)	CSFV	80.8	71	75

## Data Availability

All required data are available as texts and figures in the main text of the article. The sequence data generated in this study were submitted to GenBank and are available under Accession Numbers ON227056 and ON227057.
